# Contrasting effects of plant inter- and intraspecific variation on community trait responses to nitrogen addition and drought in typical and meadow steppes

**DOI:** 10.1186/s12870-022-03486-z

**Published:** 2022-03-01

**Authors:** Aixia Guo, Xiaoan Zuo, Senxi Zhang, Ya Hu, Ping Yue, Peng Lv, Xiangyun Li, Shenglong Zhao, Qiang Yu

**Affiliations:** 1 Urat Desert-Grassland Research Station, Northwest Institute of Eco-Environment and Resources, Chinese Academy of Sciences, Lanzhou, 730000 China; 2 University of Chinese Academy of Sciences, Beijing, 100049 China; 3 Key Laboratory of Stress Physiology and Ecology in Cold and Arid Regions, Gansu Province, Lanzhou, 730000 China; 4 Naiman Desertification Research Station, Northwest Institute of Eco-Environment and Resources, Chinese Academy of Sciences, Lanzhou, 730000 China; 5 National Hulunber Grassland Ecosystem Observation and Research Station, Institute of Agricultural Resources and Regional Planning, Chinese Academy of Agricultural Sciences, Beijing, 10008 China

**Keywords:** Community-weighted means, Drought, Inter- and intraspecific trait variation, Nitrogen addition, Plant functional traits, Typical and meadow steppes

## Abstract

**Background:**

Inter- and intraspecific variation in plant traits play an important role in grassland community assembly under global change scenarios. However, explorations of how these variations contribute to the responses of community traits to nitrogen (N) addition and drought in different grassland types are lacking. We measured the plant height, leaf area (LA), specific leaf area (SLA), leaf dry matter content (LDMC), leaf N content (LNC) and the ratio of leaf carbon (C) to leaf N (C:N) in a typical and a meadow steppe after three years of N addition, drought and their interaction. We determined the community-weighted means (CWMs) of the six traits to quantify the relative contribution of inter- and intraspecific variation to the responses of community traits to N addition and drought in the two steppes.

**Results:**

The communities in the two steppes responded to N addition and the interaction by increasing the CWM of LNC and decreasing C:N. The community in the meadow steppe responded to drought through increased CWM of LNC and reduced C:N. Significant differences were observed in SLA, LDMC, LNC and C:N between the two steppes under different treatments. The SLA and LNC of the community in the meadow steppe were greater than those of the typical steppe, and the LDMC and C:N exhibited the opposite results. Moreover, variation in community traits in the typical steppe in response to N addition and drought was caused by intraspecific variation. In contrast, the shifts in community traits in the meadow steppe in response to N addition and drought were influenced by both inter- and intraspecific variation.

**Conclusions:**

The results demonstrate that intraspecific variation contributed more to community functional shifts in the typical steppe than in the meadow steppe. Intraspecific variation should be considered to understand better and predict the response of typical steppe communities to global changes. The minor effects of interspecific variation on meadow steppe communities in response to environmental changes also should not be neglected.

**Supplementary Information:**

The online version contains supplementary material available at 10.1186/s12870-022-03486-z.

## Background

Plant functional traits play a crucial role in moderating the response of plant communities to global environmental changes [1–3]. Functional traits, i.e., measurable characteristics that directly or indirectly affect the species performance and related ecosystem properties in a particular environment [4, 5], provide essential insights for estimating ecosystem alterations in response to changing environments [6, 7]. The effects of a changing environment on community functional traits are maintained by interspecific variation, such as migration or species turnover, and by processes that contribute to the intraspecific variability in traits, such as variability in genotypic compositions or phenotypic plasticity [8]. Several studies have found that the shifts of community functional composition are mainly generated by interspecific or intraspecific trait variation or a combination of the two [8–14]. Examining the relative importance of inter- and intraspecific variation in plant community responses to environmental change is thus essential for understanding plant community assembly [12].

The relative contribution of inter- and intraspecific variation to changes in the characteristics of communities following global climate change reflect the plant community resistance to these environmental changes [15]. An increase in interspecific variation indicates greater replacement of species and decreased resistance of the composition of the community to changes in the environment [16, 17]. In turn, higher intraspecific variation primarily represents stronger resistance and adaptation of plant communities to environmental changes [14]. How do inter- and intraspecific trait variability mediate community functional responses to environmental changes depends on environmental context [18]. Some studies have predicted that extensive long-term changes to the environment may result in functional replacement through interspecific variation [19]. Variation in community traits along large environmental gradients should be caused by interspecific variation [12, 19, 20] because intraspecific variation would saturate once the entire potential genetic variation of the species is reached [21]. Conversely, moderate changes in community traits that act on shorter time scales may be affected by intraspecific variation [22]. In addition, the relative contribution of intraspecific variation is expected to increase with decreasing spatial gradient, and if there is low environmental heterogeneity, one can expect that intraspecific variation would dominate over interspecific variation [21]. Previous work suggested that trait variation was caused by interspecific variation as they involve a system of higher species richness and beta diversity, the intraspecific variation is thus more important in species-poor study systems [19].

Plant height, leaf area (LA), specific leaf area (SLA), leaf dry matter content (LDMC), leaf N content (LNC) and the ratio of leaf carbon to leaf N (C:N) are important functional traits linking the plant to the environment [23]. Plant height is related to light acquisition and competitive ability [24]. LA is associated with stress tolerance; plants tend to have smaller leaves in response to stresses [25]. SLA relates to plant growth and photosynthetic rates. Increased plant height, LA and SLA reflect rapid resource-acquisition strategy and growth rates [26]. Plants with higher LDMC have thicker and more rigid cell walls, which allows to lower leaf water potential and to maintain the cell turgor to enhance leaf resistance to physical stress [27]. Plants with high LNC have strong photosynthetic capacity and fast plant growth [28]. A decrease in the C:N reflects the high N use efficiency by plants [29]. Community-weighted mean (CWM) traits depend on the plant community composition and are related to ecosystem function, but may also be related to environmental drivers such as N addition and drought [30, 31]. Community response to environmental changes can vary greatly among different study sites. Research has shown community means SLA and LDMC decreased with increasing aridity [3]. Another study instead showed that the drought caused an increase in SLA and LDMC [32]. N addition increased community N concentration and decreased C:N ratio, but did not alter in SLA and LDMC [33]. N enrichment also cause an increase in community mean SLA and LNC and a decrease in LDMC [34].

Grasslands occupy more than 40% of the terrestrial ecosystems of China [35, 36]. Approximately 78% of the grasslands in China occur in the northern temperate region. Typical steppe and meadow steppe are two important grassland ecosystem types on the Inner Mongolian Plateau [36]. Steppe ecosystem represents relatively sensitive and vulnerable ecosystem under current global changes [37]. Considering grassland types will help assess community trait responses to global changes since different grasslands may show different response patterns [38]. For example, in typical steppe ecosystems, the responses of community chemical characteristics to N and water enrichment are mainly dominated by intraspecific variation [25]. Interspecific variation plays a significant role in driving the functional responses of community to N addition in the meadow steppe [39]. However, little is known about the relative contribution of inter- and intraspecific variation to the functional composition of communities in response to N addition and drought between the typical and meadow steppes.

Here, we focus on six key plant functional traits, i.e., plant height, LA, SLA, LDMC, LNC and the C:N ratio, related to plant resource acquisition and use strategies [15, 16]. A major aim of our study is to demonstrate how N addition and drought shape the variation in each trait and in turn, reveal the role of inter- and intraspecific trait variation in modifying community functional composition in response to N addition and drought in a typical and meadow steppe. We hypothesized that the relative importance of inter- and intraspecific variation in the plant community trait responses to N addition and drought would differ between steppe types.

## Results

Plant functional traits significantly differed among the four dominant species in the typical steppe (Figures S1-S3). *Stipa grandis* had higher height, LDMC and C:N, whereas *Agropyron cristatum* showed lower height, LDMC and C:N. Higher LA, SLA and LNC were found in *Achnatherum sibiricum* and *Leymus chinensis*, while lower LA, SLA and LNC were found in *Stipa grandis*. Both N addition and drought significantly decreased the height of *S. grandis* and LA of *Leymus chinensis* and N addition significantly increased LA of *A. cristatum* (Figure S1). Both N addition and drought enhanced SLA of *A. sibiricum* and reduced LDMC of *A. sibiricum* and *A. cristatum*, respectively (Figure S2). N addition and the combination of N addition and drought increased LNC and decreased C:N of four dominant species, while drought increased LNC and decreased C: N of *S. grandis* and *A. cristatum* (Figure S3).

In the meadow steppe, significant differences were also observed for the plant functional traits between species across different treatments (Figures S4-S6). *Leymus chinensis* had higher height and LA. *Thalictrum aquilegifolium* had higher SLA and LNC, and lower LA and C:N. *Stipa baicalensis* showed greater LDMC and lower LA and SLA. *Carex korshinskyi* exhibited larger C:N, as well as lower height and LNC. Higher LA and lower C:N were found in *Achnatherum sibiricum*. Higher SLA and LNC, and lower LDMC were found in *Artemisia tanacetifolia*. Different treatments did not affect the height of dominant species. Drought enhanced LA of *L. chinensis* while reduced LDMC of *S. baicalensis* (Figure S4). The combination of N addition and drought significantly increased SLA and decreased LDMC of *T. aquilegifolium* and *A. tanacetifolia* (Figure S5). N addition, drought and both cause an increase in LNC and a decrease in C:N of all dominant species (Figure S6).

As shown in Table 1, the site significantly affected the CWMs of LA, SLA, LDMC, LNC and C:N (*p* < 0.05). N addition and drought significantly affected the CWMs of LNC and C:N (*p* < 0.001). The interaction of site and N addition and that of site and drought significantly affected the CWM of LNC (*p* < 0.01).

**Table 1:** Results (*F* value) of three-way analysis of variance (ANOVA) on the effects of the site, N addition and drought (D) on the CWM of traits in a typical and meadow steppe. The asterisk was shown as the significance (*, *p* < 0.05; **, *p* < 0.01; ***, *p* < 0.001)

Source	Plant height	LA	SLA	LDMC	LNC	C:N
Site	0.004	5.656*	58.585***	144.613***	150.135***	209.591***
N	0.073	0.405	1.691	2.244	38.060***	48.572***
D	4.032	0.004	0.313	0.048	17.493***	18.717***
Site*N	2.364	0.652	0.628	0.01	7.611**	0.018
Site*D	1.591	2.145	0.927	0.362	10.160**	3.865
N*D	0.937	0.032	1.19	0.001	1.334	3.258
Site*N*D	0.565	0.026	0.000	1.313	0.218	0.149

**Fig. 1:**
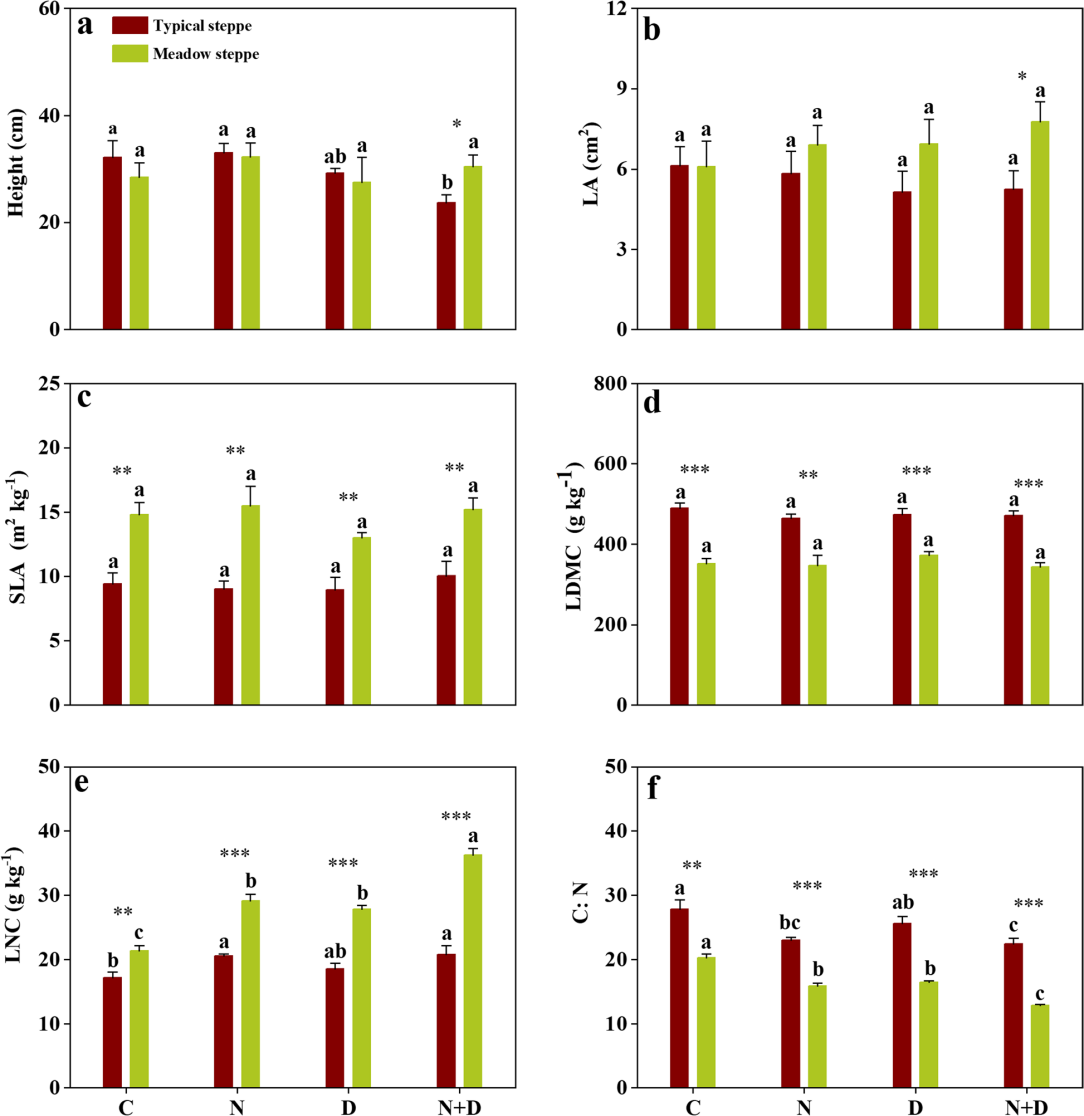
Effects of N addition and drought on the CWMs of plant functional traits in the two steppes. The values are presented as the means ± standard errors (*n* = 6). C, control; D, drought; N, N addition; N + D, both N addition and drought. Different lowercase letters for the mean values indicate significant differences among the different treatments at *p* < 0.05. Significant differences between the two steppes are reported from ANOVA as * *p* < 0.05, **p < 0.01 and ***p < 0.001

Compared with the control treatment (natural precipitation), drought increased LNC and reduced C:N in the meadow steppe but did not affect LNC and C:N in the typical steppe (Fig. 1e, f, *p* < 0.05). N addition, and both N addition and drought increased LNC and decreased C:N for both steppes (Fig. 1e, f; *p* < 0.05). Overall, N addition, drought, and both N addition and drought had no apparent impacts on LA, SLA, and LDMC in either steppe (Fig. 1b, c, d; *p* > 0.05). Notably, there were significant differences in SLA, LDMC, LNC and C:N between the two steppes under different treatments. The meadow steppe community had higher SLA and LNC than the typical steppe community did. In contrast, the meadow steppe community had lower LDMC and C:N than the typical steppe community did. In addition, there were differences in plant height and LA between the two steppe communities in response to drought and N addition interaction effects. (Fig. 1a, b; *p* < 0.05).

**Fig. 2:**
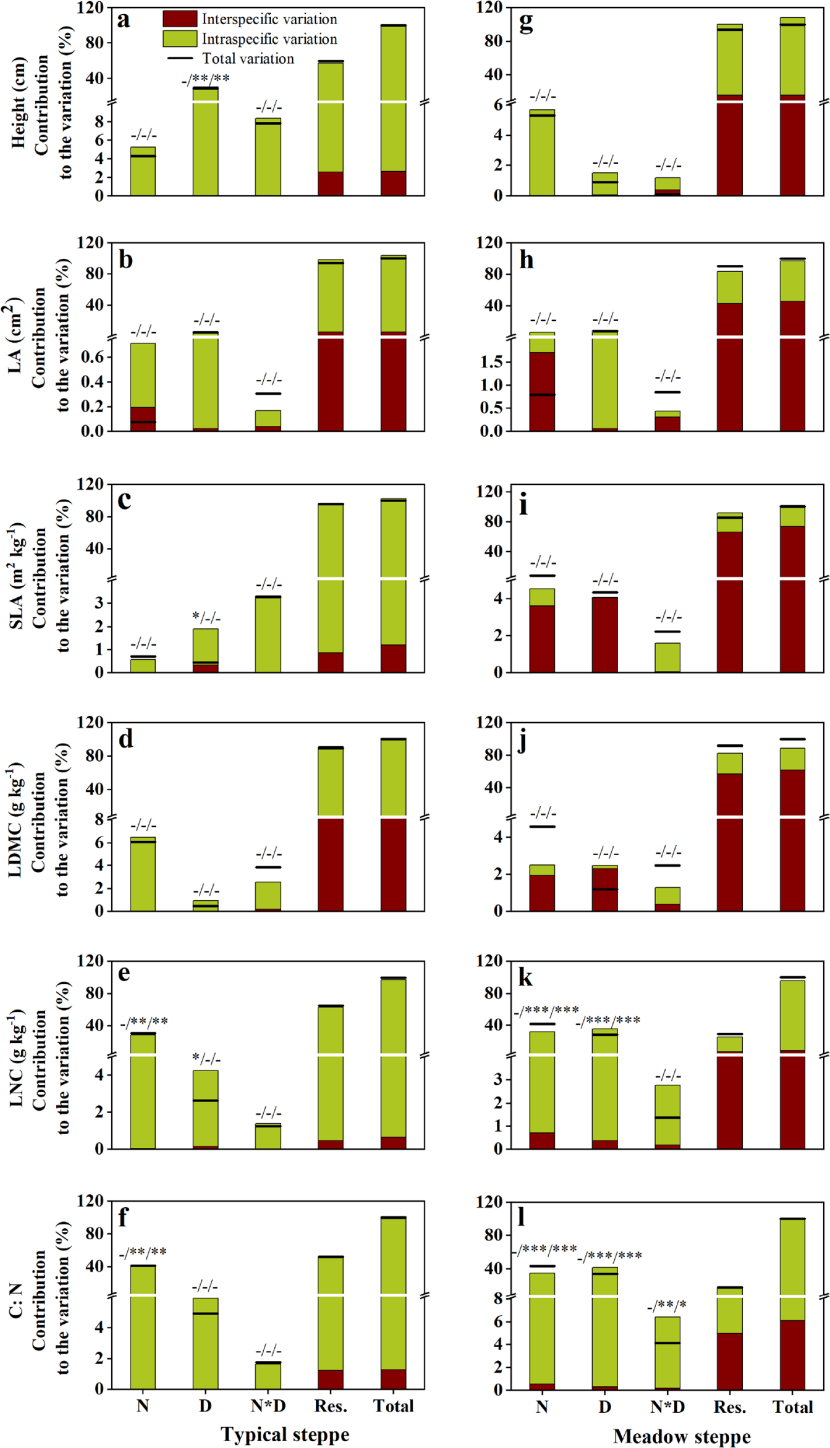
Decomposition of changes in plant functional traits using CWMs in a typical and a meadow steppe. Black bars represent total variation. The space between the top of the column and the bar corresponds to the covariation effects; positive covariation implies that the bar is above the column, and negative covariation indicates that the bar crosses the column. N, N addition; D, drought; N*D, interaction between N addition and drought; Res., Residual; Total variation, interspecific variation + interspecific variation + covariation. Significance values are indicated by an asterisk above each column. The positioning of an asterisk within the -/-/- graphic represents the significance of the interspecific variation/intraspecific variation/total variation, respectively. -, *p* > 0.05; *, *p* < 0.05; **, *p* < 0.01; ***, *p* < 0.001

In the typical steppe, the total variation in the CWM of all plant functional traits was dominantly driven by intraspecific variation. Intraspecific variation accounted for more than 92% of the total variation in all traits, and the relative contribution of interspecific variation was 0.6–8% of the variability. Under N addition, drought and their interaction, intraspecific variation contributed more to the responses of typical steppe communities to N addition and drought than did interspecific variation for all traits (Fig. 2a, b, c, d, e, f). The variability in height can be well explained by drought treatment, and the variations in LNC and C: N can be well explained by N addition, with a low level of unexplained variability (Fig. 2a, e, f).

In the meadow steppe, intraspecific variation drove the total variation in height, LA, LNC and C: N (Fig. 2g, h, k, l). The total variability in SLA and LDMC was primarily reflected by interspecific variation (74% and 62% of the variability) (Fig. 2i, j). Under the three treatments, the relative contribution of intraspecific variation in height, LNC and C: N was higher than that of interspecific variation (Fig. 2g, k, l). The relative importance of inter- and intraspecific variation in LA, SLA and LDMC varied depending on the treatments considered. Intraspecific variation made a more significant contribution to variation in LA under N addition and drought and interspecific variation under their interaction. For SLA and LDMC, interspecific variation drove their variation under N addition and drought, but intraspecific variation drove their variation under their interaction (Fig. 2i, j). The variations in LNC and C:N can be well explained by N addition, drought and their interaction, with the unexplained variability being low (Fig. 2k, l).

## Discussion

Trait-based methods have made considerable progress in understanding and predicting changes in the structure of plant communities in response to global climate changes [10, 22, 33, 40]. Environments filter individuals deterministically based on their functional traits. Therefore, environmental changes in space cause shifts in the composition of community traits, as numerous studies have shown the relationships of environment-community mean traits [4, 41, 42]. In this study, we uncovered changes in CWMs for most of the traits, and the contribution of inter- and intraspecific variation. N addition increased LNC and decreased C: N at both species- and community-level in the two steppes, indicating that N addition enhanced the availability of N nutrients and in turn reflected a shift towards a rapid resource absorption and growth strategy [43], and a decrease in the C:N is results from elevated LNC and a relatively stable carbon level under N addition [44]. Moreover, drought caused an increase in LNC of the meadow steppe, supporting the finding that suggested increased LNC with increasing drought [45]. The failure of the typical steppe community to respond to drought suggests that plant communities in different habitats have different adaptation strategies to drought, and steppe communities at the wet site have a competitive advantage to drought by increasing LNC [46, 47]. Most importantly, higher SLA in the meadow steppe than typical steppe is agreed with previous findings which suggest that SLA is usually lower in dry than in wet habitats partly because low SLA may promote tolerance to cell collapse that is induced by low water potential [48]. Plant with a higher LDMC in the typical steppe are better able to survive drought conditions because of higher resistance to physical damage by desiccation [49]. The LNC of meadow steppe is higher than that of the typical steppe, mainly because the former has higher above-ground biomass and vegetation cover, thus capturing more light resources and increasing LNC. Overall, the lack of change in community mean LA suggests that the environmental gradient in this experiment is not related to the function of LA (i.e., LA showed a neutral response).

Environmental pressure largely drives shifts in traits of the plant community, either interspecifically or intraspecifically, such that it shapes the community functional composition. An increasing body of evidence has suggested that intraspecific trait variability plays a larger role in the functional responses of the plant community to resource availability [8, 15, 16, 50, 51]. Variation in community means traits such as height, SLA and LDMC were mainly caused by intraspecific variation [52]. Comparative study also found that the relative importance of inter- and intraspecific variation in plant communities differs among leaf traits [53], and leaf nutrient concentrations and ratios are highly labile and sensitive to environment than morphological traits and therefore show higher intraspecific variation at the community level [25, 34, 54]. The whole plant traits (e.g. plant height) also display high intraspecific variation due to local genetic adaptation and phenotypic plasticity [55]. The results of this study showed that variability of community means height, LNC and C:N for both steppes in response to N addition and drought was mainly driven by intraspecific variation, while the variability of community means SLA and LDMC for the meadow steppe in response to N addition and drought was mostly caused by interspecific variation. These results suggest that the environmental gradients in this experiment were irrelevant to height, LNC and C:N, or the gradients are not steep enough to induce significant variation in these three traits. Instead, SLA and LDMC can well indicate changes in intra- and interspecific variation even under low environmental heterogeneity, suggesting that these two traits could serve as better predictors of intra- and interspecific variation along short environmental gradients.

These results agree with our hypothesis that the relative importance of inter- and intraspecific variation differs among steppe types. Community mean traits in the typical steppe respond to N addition and drought mainly through intraspecific variation. Both inter- and intraspecific variation determined variation in community mean traits in the meadow steppe. The differences between the two steppes are mainly due to shifts in community composition caused by environmental changes in space [15]. Hence, quantifying the beta diversity of environmental gradients is important to compare the results across steppes [19]. The relative importance of intraspecific variation varies among communities differing in beta diversity and decreased with increasing beta diversity [17, 56]. Community composition in the typical steppe with lower beta diversity shows low spatial variation or high resistance. This means that generalized species with high intraspecific trait variation occupy many local habitats, leaving little room for specialized species to survive. There is therefore a suppression for interspecific variation [18]. On the contrary, community trait variation in the meadow steppe would be dominated by interspecific variation since plant communities in this system have high beta diversity and species richness.

## Conclusions

Our study reveals the relative importance of inter- and intraspecific variation in the responses of community functional traits to N addition and drought in the typical and meadow steppes. Intraspecific variation plays a more significant role in explaining changes in community functional traits in the two steppes in response to N addition and drought, especially variation in traits related to nutrition, which mainly varied in response to intraspecific variation. It suggests that they have high sensitivity to N addition and to drought and shows that these traits allow the two steppe communities to respond to environmental changes. Intraspecific variation likely has a considerable impact on ecosystems and should be explicitly incorporated to predict the potential response of plant communities to future global change via a trait-based approach. However, taking the role of the interspecific variation effect into consideration in different grassland types is an essential step. Integrating inter- and intraspecific variation in trait ecology may enhance understanding processes operating at the community level. Moreover, multiple site experiments are needed to understand better and predict how community functional traits respond to global change factors.

## Materials and methods

### Experimental sites

This study was conducted at two sites across Inner Mongolia, China. (1) The first site was at the Grassland Ecosystem Research Station, the Chinese Academy of Sciences (43°38' N, 116°42' E), with an average elevation of 1,350 m and a mean annual temperature of 2.3 ℃. In this area, the climate is semiarid, temperate and continental. The mean annual precipitation (MAP) is ~ 350 mm, most of which (70%) occurs from May to September. The dominant vegetation comprises the typical steppe vegetation of the Inner Mongolia Plateau. (2) The second site was at the National Hulunber Grassland Ecosystem Observation and Research Station (49°19'- 21' N, 119°55'-58' E), with an average elevation of 610 m and a mean annual temperature from -2 ℃ to 1 ℃. The study area falls within a semihumid, continental, temperate steppe climate zone. The MAP ranges from 380 to 400 mm, most of which (80%) falls during the plant growing season between July and September. The dominant vegetation comprises the meadow steppe vegetation of the Inner Mongolia Plateau. The two sites were not affected by grazing or other anthropogenic disturbances.

### Experimental design and sampling

The experiment was set up in May 2017 following a random block design in which there were 6 blocks, with each block having 4 treatments: C, control; N, N addition; D, drought; and N + D, both N addition and drought. In total, there were 24 plots, each of which was 6 m × 6 m. N fertilizer was applied each year by applying dry-resin-coated urea (44% pure N) at a yearly rate of 10 g N m^−2^ yr^−1^. Drought-stricken plots were created by excluding 50% of natural precipitation with an automatically controlled plastic roof covering the vegetation. Water barriers with a depth of 30 cm were added belowground to each plot.

All surveys and samplings were conducted during the peak of growth in mid-August 2019. One quadrat of 1 m × 1 m was established in each plot to harvest the aboveground biomass of the plant communities. After collecting all living biomass in each quadrat, it was separated for plant species, oven-dried at 65 ℃ for 48 h to a constant mass, and weighed. Leaves of each species were collected in another quadrat to measure their height and functional traits. Twenty-nine species in the typical steppe and thirty-eight species in the meadow steppe were encountered within 24 quadrats. In the typical steppe, dominant species were *Stipa grandis*, *Achnatherum sibiricum*, *Leymus chinensis* and *Agropyron cristatum*, which relative biomass accounted for 30%, 21%, 14% and 11%, respectively. In the meadow steppe, dominant species were *Leymus chinensis*, *Thalictrum aquilegifolium*, *Stipa baicalensis*, *Carex korshinskyi*, *Achnatherum sibiricum* and *Artemisia tanacetifolia*, which relative biomass accounted for 34%, 18%, 5%, 7%, 10% and 14%, respectively. In addition, beta diversity was calculated as the ratio of the total number of species represented to the average species number across quadrats to compare the community composition of the two steppes [57]. The beta diversity of the typical steppe was lower than that of the meadow steppe.

The plant materials used in this study were obtained from the Grassland Ecosystem Research Station, the Chinese Academy of Sciences, and the National Hulunber Grassland Ecosystem Observation and Research Station. The staff at these stations permitted us to collect such materials. The details of the materials can be found via the following link: http://nmg.cern.ac.cn/ and http://hlg.cern.ac.cn/. Shurun Liu performed the formal identification of *Stipa grandis, Leymus chinensis and Agropyron cristatum*, and the deposition numbers of these plants are B0330, B0374 and B0417. Liqing Zhao and Baorui Chen undertook the formal identification of *Leymus chinensis, Thalictrum aquilegifolium, Stipa baicalensis, Carex korshinskyi, Achnatherum sibiricum and Artemisia tanacetifolia*, and the deposition numbers of these plants are HLG_ZB_2009_6045, HLG_ZB_2009_7455, HLG_ZB_2009_7102, HLG_ZB_2009_7333, HLG_ZB_2009_7101 and HLG_ZB_2009_7097. Voucher specimens of these materials have been deposited in a publicly available herbarium (herbarium of Grassland Ecosystem Research Station, the Chinese Academy of Sciences and herbarium of the National Hulunber Grassland Ecosystem Observation and Research Station). This research on wild plants and the collection of plant material complies with institutional, national, or international guidelines.

### Plant functional trait measurement

Fifteen fully expanded, healthy intact leaves from five individuals of each dominant species were randomly collected in each quadrat [58], immediately placed between two sheets of moist filter paper, and stored in self-sealing bags under refrigeration. Subsequently, they were stored in water in the dark under refrigeration at 5 °C for 12 h. Moisture on the surface of the leaves was rapidly absorbed with absorbent paper. Then, the leaves were placed on an electronic scanner [59], and LA was measured using ImageJ 1.8.0 software (https://imagej.nih.gov). Leaf fresh weight was then measured using an electronic balance. The scanned leaves were oven-dried at 65 °C for 48 h to a constant mass, and leaf dry weight was recorded. SLA was calculated as LA divided by leaf dry weight, and LDMC was calculated as leaf dry weight divided by fresh weight [60]. Then, leaves of the dominant species were collected from each plot and oven-dried at 65 °C for 48 h. The oven-dried leaves were milled and weighed to 4.5 mg to measure the leaf carbon content (LCC) and LNC. LCC and LNC were analyzed using an elemental analyser (Costech ECS 4010, Italy).

### Data analysis

The CWMs of the six functional traits were calculated in each quadrat. Each CWM was determined by using the relative biomass species as a weighting factor as follows:

$$\mathrm{CWM}=\sum {\mathrm{W}}_{\mathrm{i}}{\mathrm{X}}_{\mathrm{i}}$$

In the formula, CWM is the community-weighted mean value of a given trait; W_i_ and X_i_ are the relative biomass of the i-th species and the mean trait value of the i-th species in each plot, respectively. For each trait and plot, ‘specific’ community mean trait values were first determined by considering species-specific traits measured in each plot, including both inter- and intraspecific variation effects. The ‘interspecific’ community mean trait values were then calculated using species trait values averaged across all plots, including only the effects of interspecific variation. The ‘intraspecific’ community mean trait values were calculated as the difference between ‘specific’ and ‘interspecific’ community mean trait values [61]. The relative contribution of inter- and intraspecific variation was calculated following a previously described method [52]. The method is based on the decomposition of the total sum of squares (SS_specific_) of the community-level trait variance related to treatments (N, D and N*D) into ‘interspecific’ (SS_inter_), ‘intraspecific’ (SS_intra_) and ‘covariation’ (SS_cov_) effects, such that SS_specific_ = SS_inter_ + SS_intra_ + SS_cov_ [16, 52]. First, two-way ANOVAs was separately performed for specific, interspecific, and intraspecific community mean trait (Tables S1 and S2, *p* < 0.05). Then, the sums of squares (i.e., SSspecific, SSinter and SSintra) were extracted. Finally, the SScov part, which represents the effects of covariation between inter- and intraspecific trait variation, was obtained by subtracting SS_inter_ and SS_intra_ from SS_specific_. Positive covariation suggests that inter- and intraspecific variation reinforce each other and thus accelerate the responses of CWM trait values to treatments. Negative covariation indicates that inter- and intraspecific variation compensate for each other and thus weaken the responses of CWM trait values to treatments.

Differences in plant functional traits between treatments within species and differences in plant functional traits between species within treatments were detected with one-way ANOVA and subsequent least-significant difference (LSD, *p* < 0.05) multiple comparison post hoc test. Three-way ANOVAs was used to examine the effects of site, N addition and drought on CWM values of plant functional traits. One-way ANOVA was used to analyze the effects of N addition and drought on the CWM values of plant functional traits, and multiple comparisons were tested by LSD (*p* < 0.05). All statistical analyses were performed and graphs were generated using SPSS 22.0 software (SPSS Inc., Chicago, Illinois, USA) and Origin 9.1 software (Origin Lab, Hampton, MA, USA).

## Supplementary Information

**Additional file 1: Figure S1.** Effects of N addition and drought on the plant height and LA of four dominant species in the typical steppe. **Figure S2.** Effects of N addition and drought on the SLA and LDMC of four dominant species in the typical steppe. **Figure S3.** Effects of N addition and drought on the LNC and C:N of four dominant species in the typical steppe. **Figure S4.** Effects of N addition and drought on the plant height and LA of six dominant species in the meadow steppe. **Figure S5.** Effects of N addition and drought on the SLA and LDMC of six dominant species in the meadow steppe. **Figure S6.** Effects of N addition and drought on the LNC and C:N of six dominant species in the meadow steppe. **Table S1.** Results of two-way ANOVAs of N addition and drought effects on six community-weighted average trait values in the typical steppe. **Table S2.** Results of two-way ANOVAs of N addition and drought effects on six community-weighted average trait values in the meadow steppe.

## Data Availability

The dataset supporting the conclusions of this article are included within the article.

## Abbreviations

N: Nitrogen
LA: Leaf area
SLA: Specific leaf area
LDMC: Leaf dry matter content
LNC: Leaf N content
C:N: the ratio of leaf carbon to leaf nitrogen
CWMs: Community-weighted means
