# Supplementary material for: Contrasting effects of plant inter- and intraspecific variation on community trait responses to nitrogen addition and drought in typical and meadow steppes
Source: BMC Plant Biol. 2022 Mar 1;22:90. doi: 10.1186/s12870-022-03486-z (PMC8886796; doi:10.1186/s12870-022-03486-z)
Supplement: Supplementary file 1 — Additional file 1: Figure S1. Effects of N addition and drought on the plant height and LA of four dominant species in the typical steppe. Figure S2. Effects of N addition and drought on the SLA and LDMC of four dominant species in the typical steppe. Figure S3. Effects of N addition and drought on the LNC and C:N of four dominant species in the typical steppe. Figure S4. Effects of N addition and drought on the plant height and LA of six dominant species in the meadow steppe. Figure S5. Effects of N addition and drought on the SLA and LDMC of six dominant species in the meadow steppe. Figure S6. Effects of N addition and drought on the LNC and C:N of six dominant species in the meadow steppe. Table S1. Results of two-way ANOVAs of N addition and drought effects on six community-weighted average trait values in the typical steppe. Table S2. Results of two-way ANOVAs of N addition and drought effects on six community-weighted average trait values in the meadow steppe. [file 12870_2022_3486_MOESM1_ESM.doc]

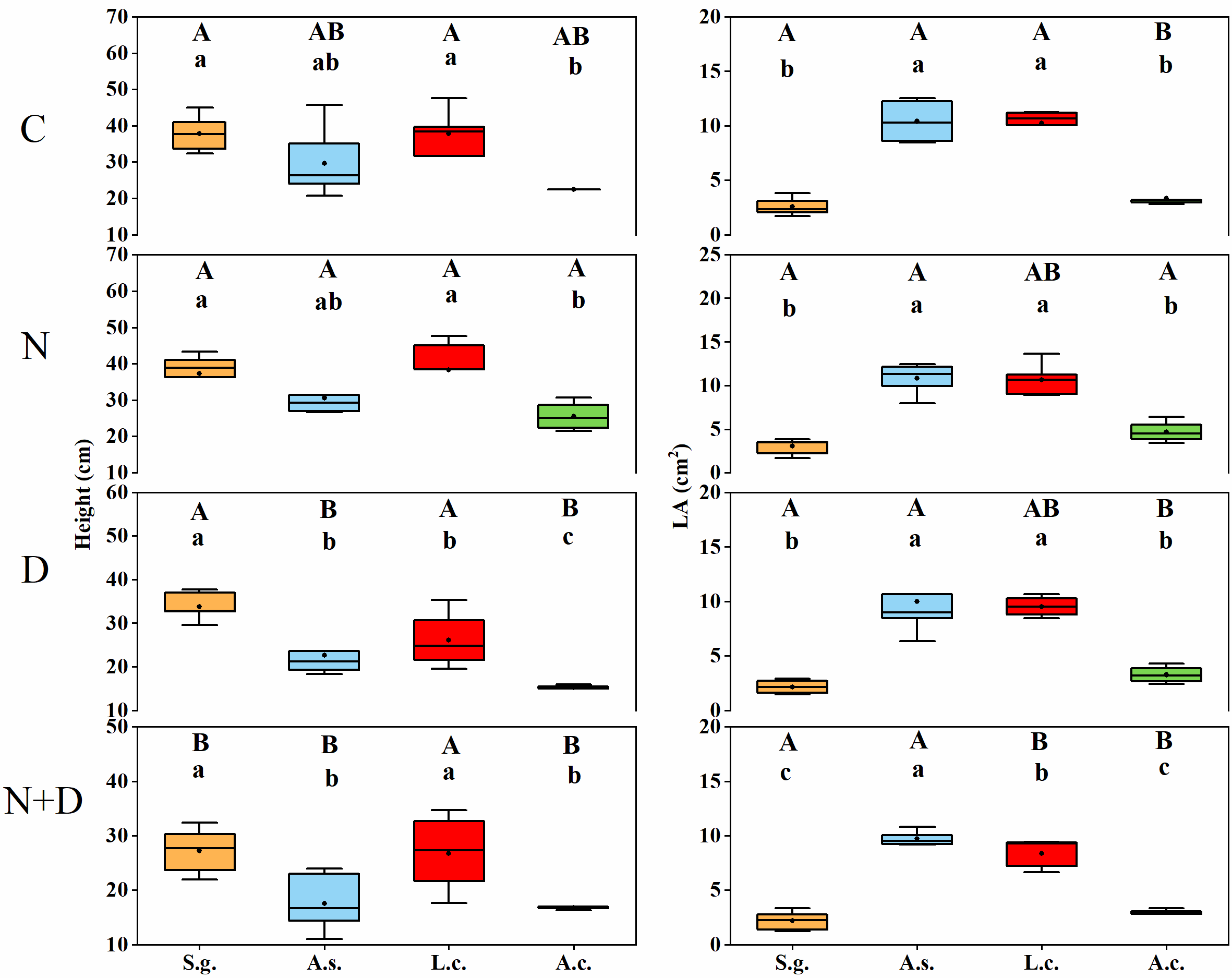


Figure S1. Effects of N addition and drought on the plant height and LA of four dominant species in the typical steppe, C, N, D and N+D (top to bottom panel). S.g., *Stipa grandis*; A.s., *Achnatherum sibiricum*; L.c., *Leymus chinensis*; A.c., *Agropyron cristatum.* Different uppercase letters indicate significant differences between different treatments for a particular species (*p* *<* 0.05). Different lowercase letters indicate significant differences between different species for a particular treatment (*p* *<* 0.05).


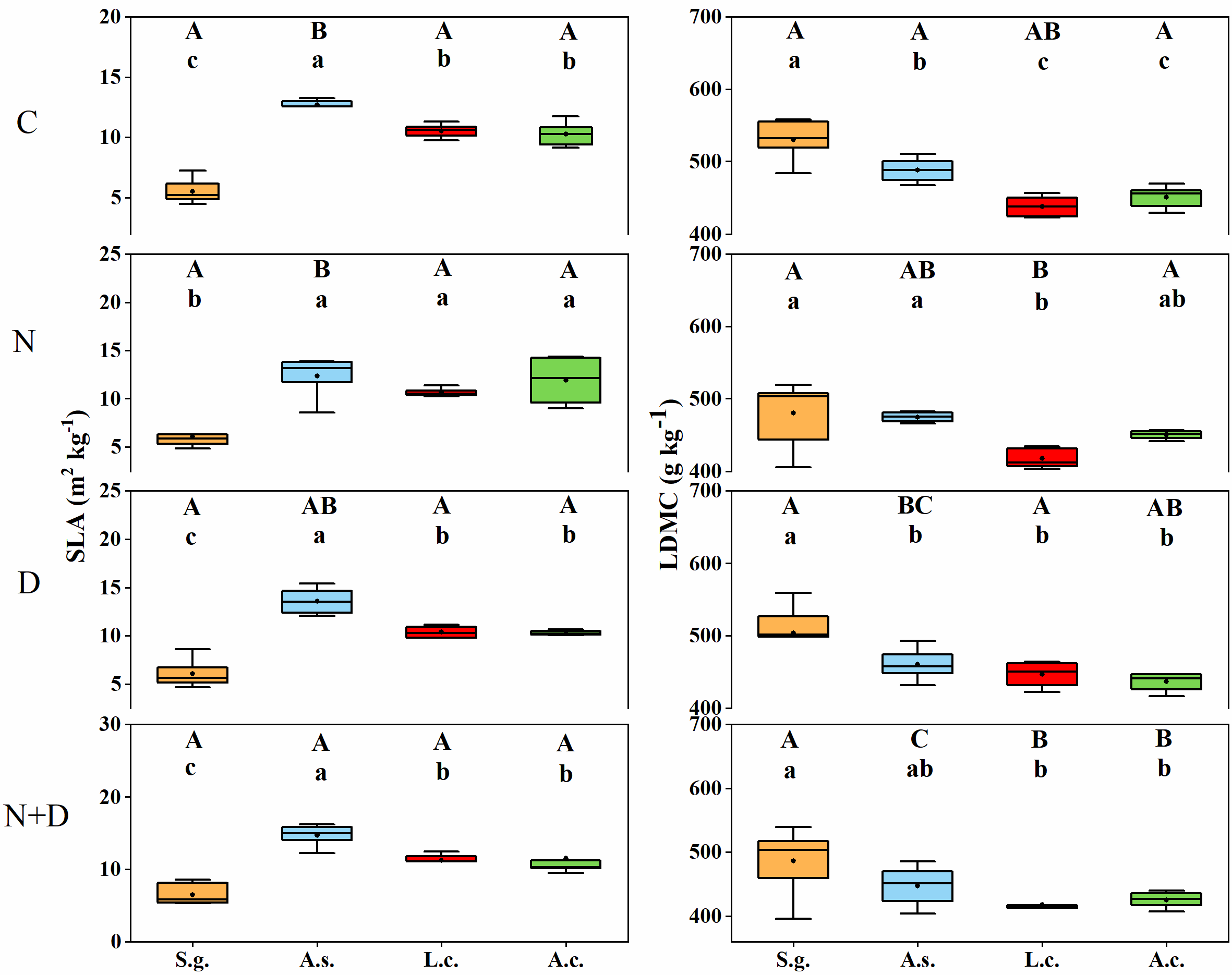


Figure S2. Effects of N addition and drought on the SLA and LDMC of four dominant species in the typical steppe, C, N, D and N+D (top to bottom panel). S.g., *Stipa grandis*; A.s., *Achnatherum sibiricum*; L.c., *Leymus chinensis*; A.c., *Agropyron cristatum.* Different uppercase letters indicate significant differences between different treatments for a particular species (*p* *<* 0.05). Different lowercase letters indicate significant differences between different species for a particular treatment (*p* *<* 0.05).


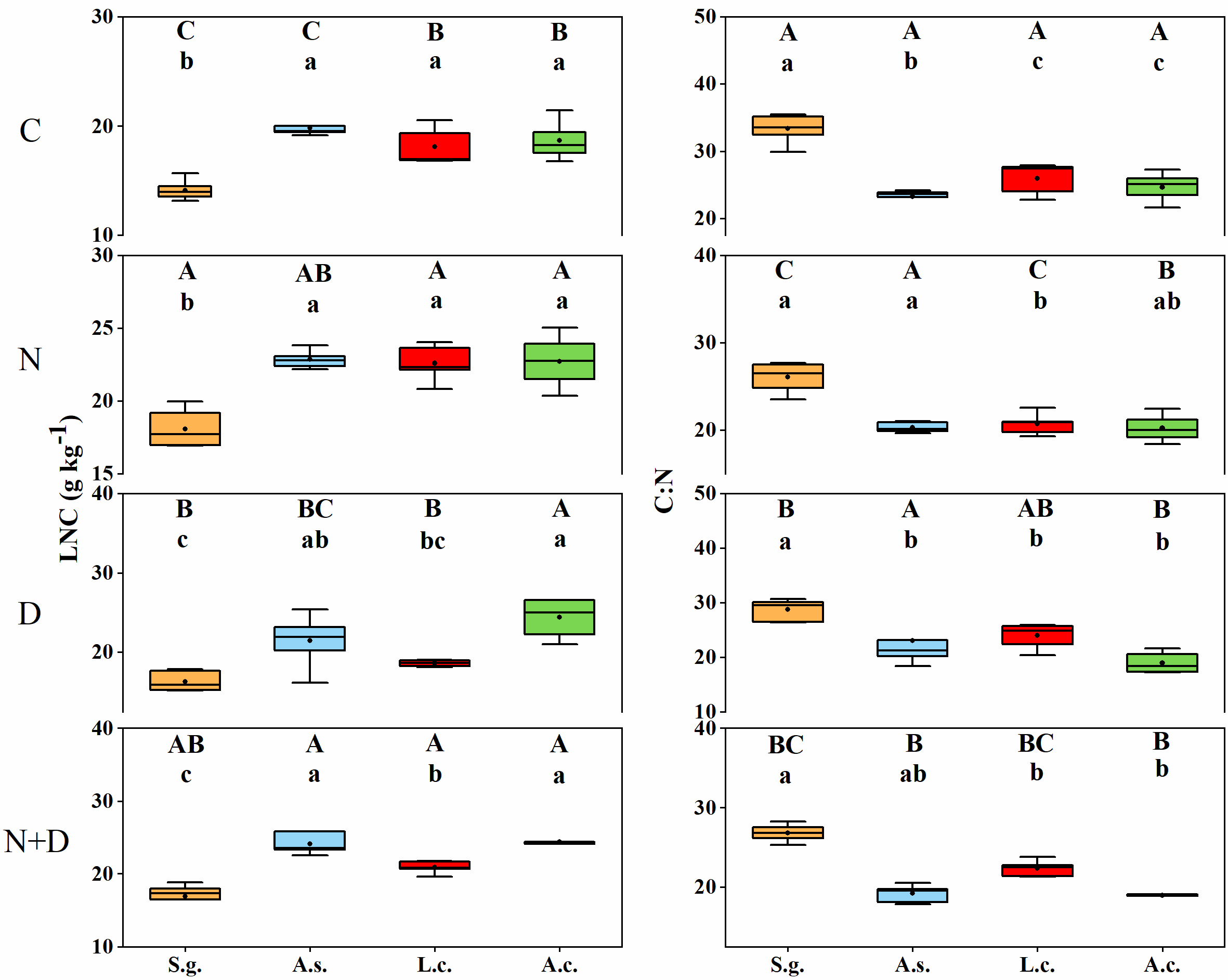


Figure S3. Effects of N addition and drought on the LNC and C:N of four dominant species in the typical steppe, C, N, D and N+D (top to bottom panel). S.g., *Stipa grandis*; A.s., *Achnatherum sibiricum*; L.c., *Leymus chinensis*; A.c., *Agropyron cristatum.* Different uppercase letters indicate significant differences between different treatments for a particular species (*p* *<* 0.05). Different lowercase letters indicate significant differences between different species for a particular treatment (*p* *<* 0.05).


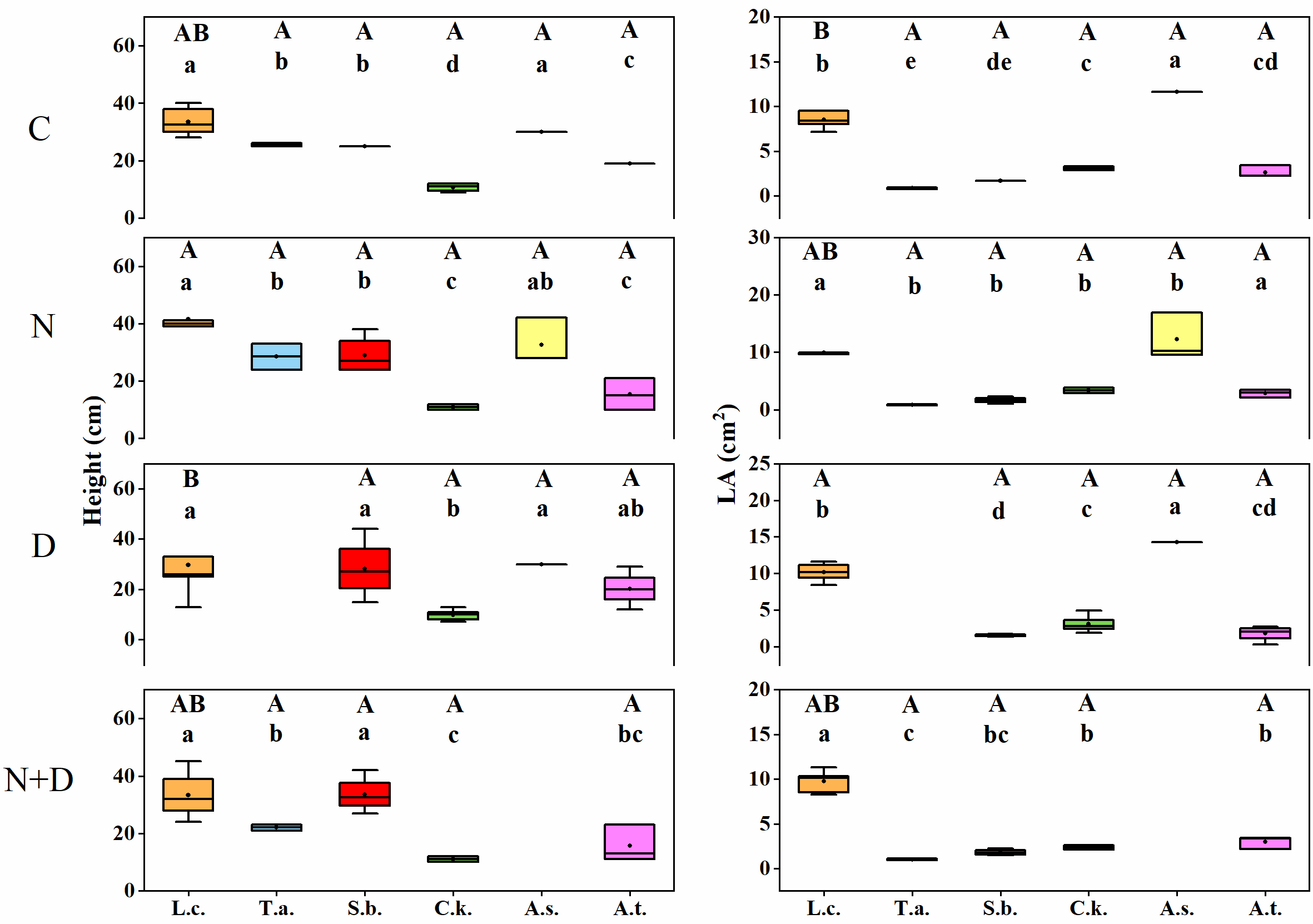


Figure S4. Effects of N addition and drought on the plant height and LA of six dominant species in the meadow steppe, C, N, D and N+D (top to bottom panel). L.c., *Leymus chinensis*; T.a., *Thalictrum aquilegifolium*; S.b., *Stipa baicalensis*; C.k., *Carex korshinskyi*; A.s., *Achnatherum sibiricum*; A.t., *Artemisia tanacetifolia*. Different uppercase letters indicate significant differences between different treatments for a particular species (*p* *<* 0.05). Different lowercase letters indicate significant differences between different species for a particular treatment (*p* *<* 0.05).


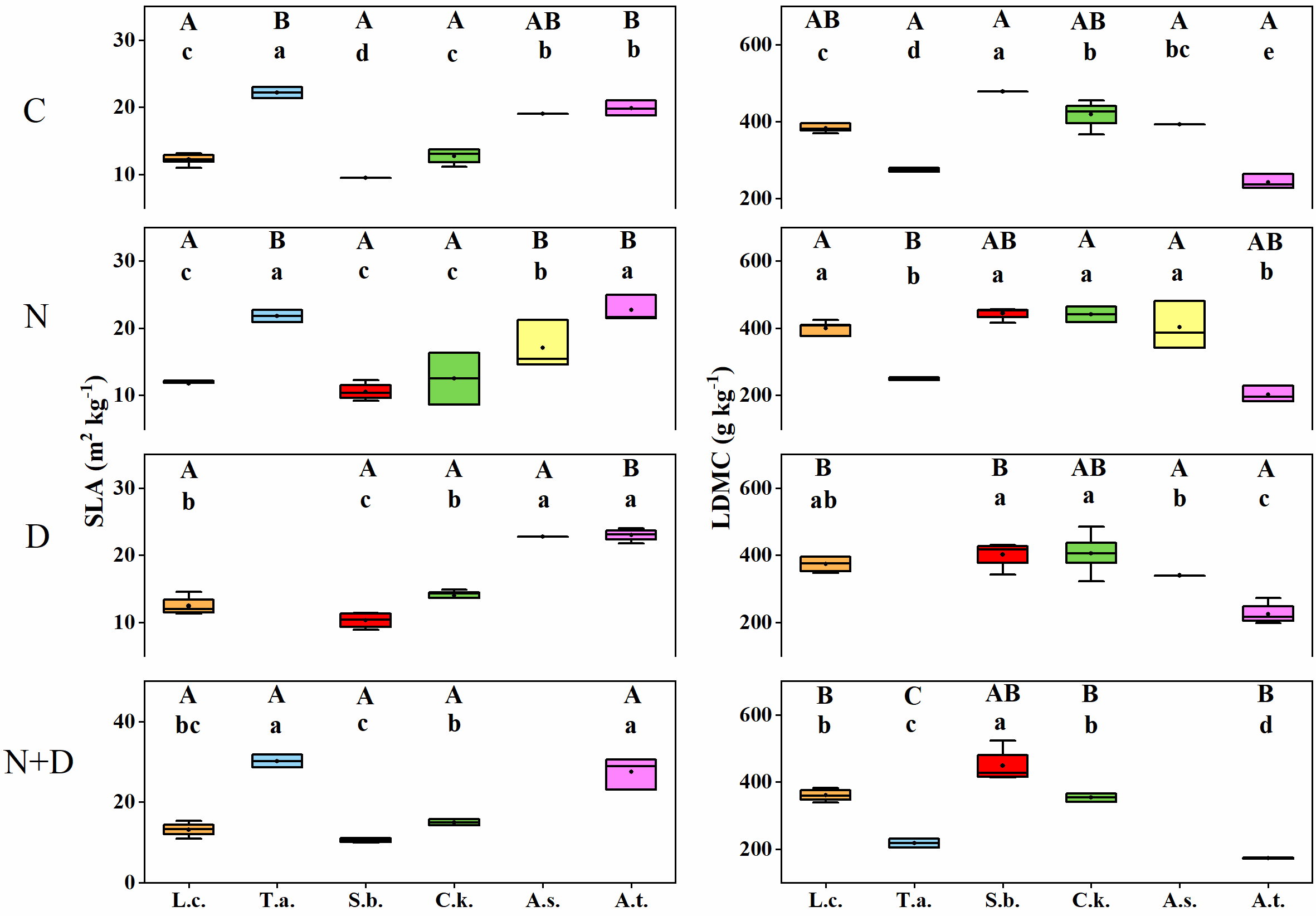


Figure S5. Effects of N addition and drought on the SLA and LDMC of six dominant species in the meadow steppe, C, N, D and N+D (top to bottom panel). L.c., *Leymus chinensis*; T.a., *Thalictrum aquilegifolium*; S.b., *Stipa baicalensis*; C.k., *Carex korshinskyi*; A.s., *Achnatherum sibiricum*; A.t., *Artemisia tanacetifolia*. Different uppercase letters indicate significant differences between different treatments for a particular species (*p* *<* 0.05). Different lowercase letters indicate significant differences between different species for a particular treatment (*p* *<* 0.05).


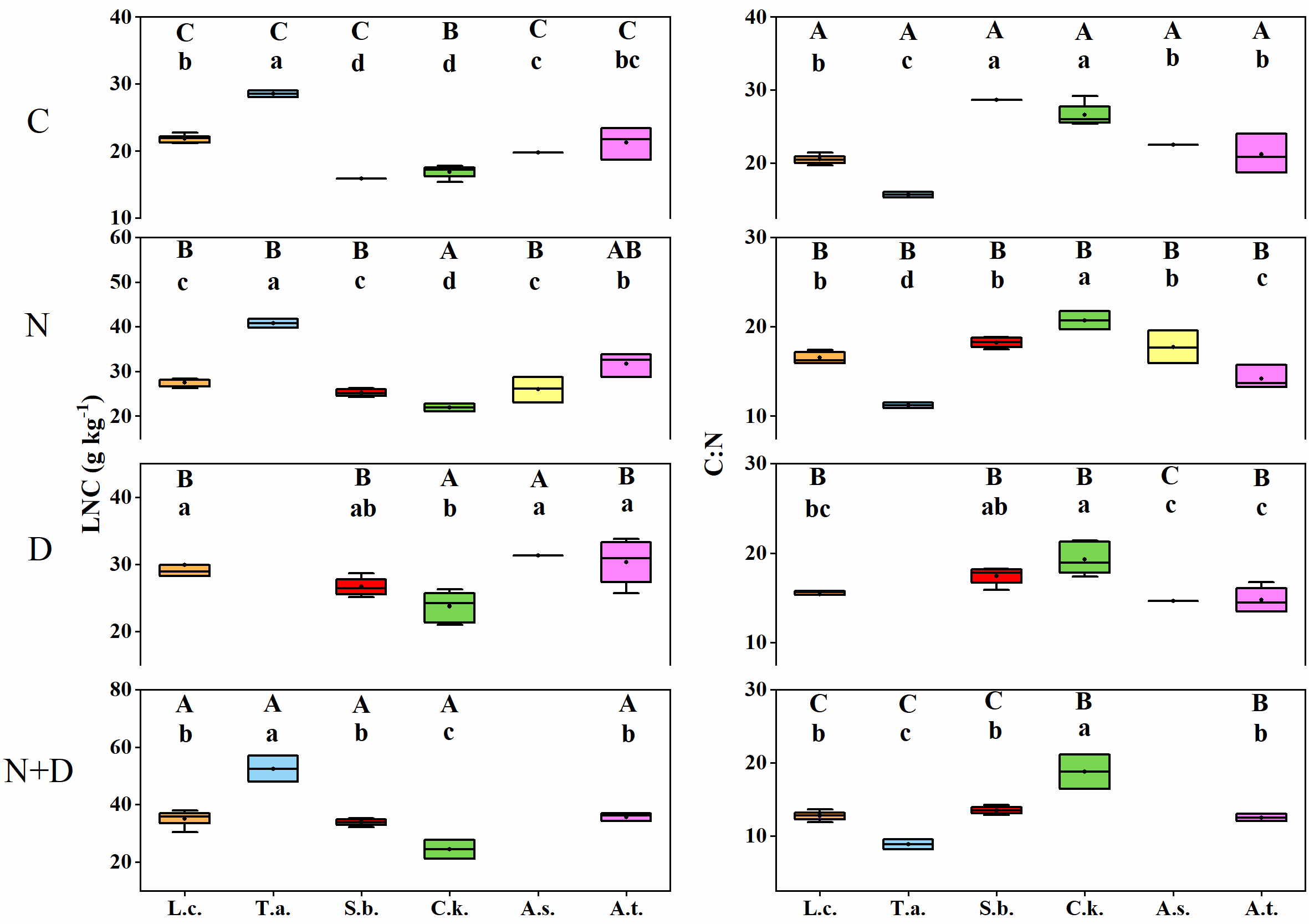


Figure S6. Effects of N addition and drought on the LNC and C:N of six dominant species in the meadow steppe, C, N, D and N+D (top to bottom panel). L.c., *Leymus chinensis*; T.a., *Thalictrum aquilegifolium*; S.b., *Stipa baicalensis*; C.k., *Carex korshinskyi*; A.s., *Achnatherum sibiricum*; A.t., *Artemisia tanacetifolia*. Different uppercase letters indicate significant differences between different treatments for a particular species (*p* *<* 0.05). Different lowercase letters indicate significant differences between different species for a particular treatment (*p* *<* 0.05).

Table S1. Results of two-way ANOVAs of N addition and drought effects on six community-weighted average trait values in the typical steppe. Interspecific, specific, and intraspecific variability effects were separately analyzed by two-way ANOVAs. The sum of squares (SS) corresponds to the amount of variability.

|  |  | Specific |  |  |  |  | Interspecific |  |  |  |  | Intraspecific |  |  |  |  |
| --- | --- | --- | --- | --- | --- | --- | --- | --- | --- | --- | --- | --- | --- | --- | --- | --- |
|  |  | SS | df | MS | F | p | SS | df | MS | F | p | SS | df | MS | F | p |
| N | Height | 33.63 | 1 | 33.63 | 1.44 | 0.244 | 0.368 | 1 | 0.368 | 0.364 | 0.553 | 41.029 | 1 | 41.029 | 1.919 | 0.181 |
|  | LA | 0.056 | 1 | 0.056 | 0.016 | 0.900 | 0.143 | 1 | 0.143 | 0.724 | 0.405 | 0.378 | 1 | 0.378 | 0.112 | 0.742 |
|  | SLA | 0.725 | 1 | 0.725 | 0.150 | 0.703 | 0.01 | 1 | 0.01 | 0.238 | 0.631 | 0.561 | 1 | 0.561 | 0.115 | 0.738 |
|  | LDMC | 1200.062 | 1 | 1200.062 | 1.357 | 0.258 | 1.251 | 1 | 1.251 | 0.015 | 0.903 | 1278.814 | 1 | 1278.814 | 1.559 | 0.226 |
|  | LNC | 46.733 | 1 | 46.733 | 9.533 | 0.006 | 0.069 | 1 | 0.069 | 2.024 | 0.170 | 43.202 | 1 | 43.202 | 9.207 | 0.007 |
|  | C:N | 97.445 | 1 | 97.445 | 15.833 | 0.001 | 0.001 | 1 | 0.001 | 0.005 | 0.946 | 96.922 | 1 | 96.922 | 15.97 | 0.001 |
| D | Height | 223.687 | 1 | 223.687 | 9.579 | 0.006 | 0.186 | 1 | 0.186 | 0.183 | 0.673 | 236.756 | 1 | 236.756 | 11.074 | 0.003 |
|  | LA | 3.760 | 1 | 3.760 | 1.094 | 0.308 | 0.017 | 1 | 0.017 | 0.084 | 0.775 | 3.278 | 1 | 3.278 | 0.969 | 0.337 |
|  | SLA | 0.457 | 1 | 0.457 | 0.094 | 0.762 | 0.341 | 1 | 0.341 | 7.801 | 0.011 | 1.586 | 1 | 1.586 | 0.326 | 0.574 |
|  | LDMC | 90.055 | 1 | 90.055 | 0.102 | 0.753 | 12.557 | 1 | 12.557 | 0.154 | 0.699 | 169.868 | 1 | 169.868 | 0.207 | 0.654 |
|  | LNC | 3.977 | 1 | 3.977 | 0.811 | 0.378 | 0.242 | 1 | 0.242 | 7.065 | 0.015 | 6.181 | 1 | 6.181 | 1.317 | 0.265 |
|  | C: N | 11.62 | 1 | 11.62 | 1.888 | 0.185 | 0.103 | 1 | 0.103 | 0.698 | 0.413 | 13.908 | 1 | 13.908 | 2.292 | 0.146 |
| N*D | Height | 61.857 | 1 | 61.857 | 2.649 | 0.119 | 0.069 | 1 | 0.069 | 0.069 | 0.796 | 66.068 | 1 | 66.068 | 3.090 | 0.094 |
|  | LA | 0.224 | 1 | 0.224 | 0.065 | 0.801 | 0.029 | 1 | 0.029 | 0.146 | 0.707 | 0.093 | 1 | 0.093 | 0.027 | 0.870 |
|  | SLA | 3.323 | 1 | 3.323 | 0.686 | 0.417 | 0.000 | 1 | 0.000 | 0.003 | 0.954 | 3.368 | 1 | 3.368 | 0.693 | 0.415 |
|  | LDMC | 765.123 | 1 | 765.123 | 0.865 | 0.363 | 36.605 | 1 | 36.605 | 0.450 | 0.510 | 467.019 | 1 | 467.019 | 0.569 | 0.459 |
|  | LNC | 1.898 | 1 | 1.898 | 0.387 | 0.541 | 0.005 | 1 | 0.005 | 0.149 | 0.704 | 2.100 | 1 | 2.100 | 0.448 | 0.511 |
|  | C:N | 4.200 | 1 | 4.200 | 0.682 | 0.418 | 0.006 | 1 | 0.006 | 0.043 | 0.838 | 3.880 | 1 | 3.880 | 0.639 | 0.433 |

Table S2. Results of two-way ANOVAs of N addition and drought effects on six community-weighted average trait values in the meadow steppe. Interspecific, specific, and intraspecific variability effects were separately analyzed by two-way ANOVAs. The sum of squares (SS) corresponds to the amount of variability.

| Treatment | Trait | Specific |  |  |  |  | Interspecific |  |  |  |  | Intraspecific |  |  |  |  |
| --- | --- | --- | --- | --- | --- | --- | --- | --- | --- | --- | --- | --- | --- | --- | --- | --- |
|  |  | SS | df | MS | F | p | SS | df | MS | F | p | SS | df | MS | F | p |
| N | Height | 68.378 | 1 | 68.378 | 1.133 | 0.300 | 0.084 | 1 | 0.084 | 0.008 | 0.928 | 73.255 | 1 | 73.255 | 1.349 | 0.259 |
|  | LA | 0.988 | 1 | 0.988 | 0.175 | 0.680 | 2.13 | 1 | 2.130 | 0.786 | 0.386 | 6.02 | 1 | 6.020 | 2.401 | 0.137 |
|  | SLA | 12.298 | 1 | 12.298 | 1.925 | 0.181 | 5.406 | 1 | 5.406 | 1.092 | 0.308 | 1.397 | 1 | 1.397 | 0.729 | 0.403 |
|  | LDMC | 1574.964 | 1 | 1574.964 | 0.998 | 0.330 | 670.878 | 1 | 670.878 | 0.682 | 0.419 | 190.013 | 1 | 190.013 | 0.435 | 0.517 |
|  | LNC | 320.251 | 1 | 320.251 | 28.673 | 0.000 | 5.597 | 1 | 5.597 | 1.981 | 0.175 | 241.174 | 1 | 241.174 | 35.189 | 0.000 |
|  | C:N | 105.253 | 1 | 105.253 | 48.092 | 0.000 | 1.378 | 1 | 1.378 | 2.283 | 0.146 | 82.548 | 1 | 82.548 | 58.200 | 0.000 |
| D | Height | 11.662 | 1 | 11.662 | 0.193 | 0.665 | 0.814 | 1 | 0.814 | 0.08 | 0.780 | 18.638 | 1 | 18.638 | 0.343 | 0.565 |
|  | LA | 9.767 | 1 | 9.767 | 1.727 | 0.204 | 0.078 | 1 | 0.078 | 0.029 | 0.867 | 8.097 | 1 | 8.097 | 3.229 | 0.087 |
|  | SLA | 6.51 | 1 | 6.51 | 1.019 | 0.325 | 6.111 | 1 | 6.111 | 1.235 | 0.280 | 0.006 | 1 | 0.006 | 0.003 | 0.955 |
|  | LDMC | 415.002 | 1 | 415.002 | 0.263 | 0.614 | 794.996 | 1 | 794.996 | 0.808 | 0.379 | 61.216 | 1 | 61.216 | 0.14 | 0.712 |
|  | LNC | 218.226 | 1 | 218.226 | 19.538 | 0.000 | 2.891 | 1 | 2.891 | 1.023 | 0.324 | 271.354 | 1 | 271.354 | 39.593 | 0.000 |
|  | C: N | 82.585 | 1 | 82.585 | 37.735 | 0.000 | 0.803 | 1 | 0.803 | 1.331 | 0.262 | 99.675 | 1 | 99.675 | 70.275 | 0.000 |
| N*D | Height | 0.98 | 1 | 0.98 | 0.016 | 0.900 | 5.023 | 1 | 5.023 | 0.495 | 0.490 | 10.441 | 1 | 10.441 | 0.192 | 0.666 |
|  | LA | 1.063 | 1 | 1.063 | 0.188 | 0.669 | 0.388 | 1 | 0.388 | 0.143 | 0.709 | 0.167 | 1 | 0.167 | 0.066 | 0.799 |
|  | SLA | 3.36 | 1 | 3.36 | 0.526 | 0.477 | 0.103 | 1 | 0.103 | 0.021 | 0.887 | 2.288 | 1 | 2.288 | 1.195 | 0.287 |
|  | LDMC | 852.518 | 1 | 852.518 | 0.54 | 0.471 | 136.756 | 1 | 136.756 | 0.139 | 0.713 | 306.378 | 1 | 306.378 | 0.702 | 0.412 |
|  | LNC | 10.574 | 1 | 10.574 | 0.947 | 0.342 | 1.465 | 1 | 1.465 | 0.519 | 0.480 | 19.911 | 1 | 19.911 | 2.905 | 0.104 |
|  | C:N | 10.01 | 1 | 10.01 | 4.574 | 0.045 | 0.496 | 1 | 0.496 | 0.822 | 0.375 | 14.963 | 1 | 14.963 | 10.549 | 0.004 |
